# Impact of Mild Oven Cooking Treatments on Carotenoids and Tocopherols of *Cheddar* and *Depurple* Cauliflower (*Brassica oleracea* L. var. botrytis)

**DOI:** 10.3390/antiox10020196

**Published:** 2021-01-29

**Authors:** Ancuta Nartea, Benedetta Fanesi, Pasquale Massimiliano Falcone, Deborah Pacetti, Natale Giuseppe Frega, Paolo Lucci

**Affiliations:** 1 Department of Agricultural, Food and Environmental Sciences, Polytechnic University of Marche, Via Brecce Bianche, 60131 Ancona, Italy; a.nartea@pm.univpm.it (A.N.); benedetta.fanesi@hotmail.it (B.F.); p.m.falcone@staff.univpm.it (P.M.F.); n.g.frega@univpm.it (N.G.F.); 2 Department of Agri-Food, Animal and Environmental Sciences, University of Udine, Via Sondrio 2/a, 33100 Udine, Italy; paolo.lucci@uniud.it

**Keywords:** boiling, carotenoids, cruciferous, steam oven, *sous-vide*, vitamin A, vitamin E

## Abstract

The effect of steam and *sous-vide* oven procedures on liposoluble antioxidants of colored cauliflower (orange and purple) was assessed for the first time and compared with domestic practice (boiling). In raw samples, the total carotenoid content was 10-fold higher in *Cheddar* than in *Depurple* (20.9 ± 2.1 vs. 2.3 ± 0.5 mg/kg dry weight), whereas the level of tocopherols was similar (28.5 ± 4.4 vs. 33 ± 5.2 mg/kg dry weight). The *Cheddar* liposoluble antioxidant matter contained violaxanthin, neoxanthin, α-carotene and δ-tocopherol, not detected in *Depurple*. All tests increased the bioactive compounds extractability with steam oven and *sous-vide* displaying similar effects, lower than boiling. In boiled *Cheddar* cauliflower, the total carotenoids and tocopherols contents increased with cooking time until they were 13-fold and 6-fold more than in raw cauliflower, respectively. Conversely, in the *Depurple* variety, contents increased by half with respect to the orange variety. However, from a nutritional point of view, no differences were revealed among the three different cooking treatments in terms of vitamin A and E levels expressed in μg/100 g of fresh vegetable because of the higher water content of boiled samples that must be considered when evaluating the effect of thermal treatment on cauliflower nutritional traits.

## 1. Introduction

Because of the growing scientific recognition that food, or food components, significantly influence human health, people have been looking for foods rich in bioactive compounds with functional effects on the prevention of nutrition-related diseases and that cause physical and mental well-being improvements.

A copious number of studies have suggested that consumption of *Brassica oleracea* vegetables, such as broccoli and cauliflower, can be associated with a decreased risk of common cancers, especially related to sulfur compounds [1,2]. Carotenoids are involved in the prevention and treatment of cardiovascular diseases [3]. The functional effects exerted by these vegetables are the result of synergistic action of a wide spectrum of phytochemicals, such vitamin C, lipid-soluble antioxidants (carotenoids, tocopherols), hydrophilic antioxidants (phenolic compounds) and glucosinolates [4]. The phytochemicals levels in cruciferous vegetables depend on different factors, mainly related to a plant’s genetic background, postharvest handling practices and technological treatments carried out after vegetable harvest. In particular, cruciferous vegetables require a cooking process before consumption that may have a positive or negative impact on their bioactive properties.

Heat treatment can induce two opposite effects on bioactive compounds concentration: a reduction of phytochemicals level as an effect of thermal degradation and/or an increase of extractability of compounds as an effect of matrix softening. The extent of the two opposite effects are simultaneously related to the structure of vegetable matrix, operative conditions and chemical nature of the specific compound [5]. For example, dos Reis et al. [6] emphasized the key role of the vegetable structure, recognizing that boiling (100 °C, 5 min) significantly increased the extractability of quercetin and kaempferol in broccoli (*Brassica oleracea* var. Avenger), whereas this strongly reduced them in cauliflower (*Brassica oleracea* var. Alphina F1). In both cases, boiled vegetables presented a lower total phenolic content and higher total carotenoid content than raw vegetable. In a similar way, a *sous-vide* cooking process accomplished in a water bath (90 °C, 20 min) increased the carotenoid availability in cauliflower and broccoli, whereas it significantly reduced the total polyphenol levels exclusively in cauliflowers. Microwave treatments led to a retention of glucosinolates in broccoli, whereas they caused a significant loss of glucosinolates in cauliflower [7].

In view of this, to achieve the beneficial effects of vegetable consumption, each vegetable should be cooked with the most suitable method able to best preserve and improve its nutritional and phytochemical value. Moreover, it is important to optimize processing parameters (i.e., temperature, time, heat transfer mode) to promote the bioactivity of the consumed vegetable and to counteract, as much as possible, undesired effects.

Emerging oven cooking processes based on mild thermal treatment of foods with minimal or rapid heating using steam as heat transfer medium, such as steam oven and *sous-vide*, are being investigated. *Brassicaceae* species are considered promising functional food ingredients, but the processing for novel food products is crucial to preserve their functionalities [8]. In this optic, mild thermal treatments could be a solution to optimize the manufacturing of foods. Their impact was recently assessed on the level of some selected antioxidants and oxidative status of salmon [9]. The results proved that steam oven treatment (65 °C, 20 min) was more efficient in enhancing the availability of the most important antioxidant compounds in salmon, such as the astaxanthin, than traditional convection oven process (180 °C, 20 min). Pellegrini et al. [7] noticed that the steam oven method (100 °C, 13 min) had a favorable effect on both fresh broccoli and white cauliflower, determining an increase of their glucosinolates total contents. Conversely, boiling treatment led to a retention of glucosinolates in broccoli, whereas they determined an important loss of glucosinolates in cauliflower. Florkiewicz et al. [10] emphasized that the use of the *sous-vide* method (90 °C, 45 min) was particularly disadvantageous and reduced the total content of glucosinolates in Romanesco-type cauliflower, whereas it enhanced the glucosinolates levels in broccoli.

Starting from this premise, in the present study, the impact of mild (steam- and *sous-vide*) oven cooking treatments on carotenoid and tocopherol profiles of colored cauliflowers (*Brassica oleracea* L. var. botrytis) was evaluated and compared with that of traditional domestic practices, such as boiling. In addition, the effect of cooking time on carotenoids and tocopherols concentrations was assessed in each tested cooking procedure. The evolution of these compounds was monitored in samples treated with the same temperature for different cooking times.

The present study represents a first attempt to evaluate the impact of innovative mild oven treatments on lipid soluble antioxidant compounds of emerging colored cauliflowers, such *Cheddar* (orange) and *Depurple* (purple). In fact, most previous research has focused exclusively on the effects of steam oven and *sous-vide* on polyphenols and glucosinolates of white and green cauliflowers. Moreover, in the present study, for a first time, was outlined the carotenoid and tocopherol profile of purple cauliflowers cv *Depurple*. Although these compounds were recently investigated in purple cauliflower cv *Graffiti* [11,12,13], no data was found concerning the *Depurple* variety.

Overall, the study contributes to evaluate if mild oven technologies could represent a valuable alternative to traditional techniques for cauliflower processing. The outcomes of this study could help the food industry, as well as the consumers, in selecting the most suitable cooking process and time able to best preserve and improve the nutritional properties and liposoluble antioxidants availability in emerging colored cauliflowers, such as *Cheddar* (orange) and *Depurple* (purple).

## 2. Materials and Methods

### 2.1. Chemicals and Reagents

Carotenoids standards (>95% purity; neoxanthin, violaxanthin, lutein, α-carotene, β-carotene), tocopherols standards (>95% purity, α-tocopherol, γ-tocopherol and δ-tocopherol), solvents HPLC grade (>95% purity, acetone, acetonitrile, dichloromethane, ethanol, methanol, *n*-hexane, isopropanol, acetic acid and water), ammonium acetate (HPLC grade, >99%) and ascorbic acid (>99.5%) were purchased by Merck (Darmstadt, Germany). Potassium hydroxide (85%) and anhydrous sodium sulphate were purchased by ITW Company (Darmstadt, Germany). MilliQ water was purified with Millipure System (Millford, SC, USA).

### 2.2. Cauliflower Samples

In total, 6 kg of cauliflowers, *Cheddar* and *Depurple* varieties, were provided by a local company (Agrinovana S.r.l Petritoli, Fermo, Italy). The cauliflower rosettes were removed of damaged tissue, leaves and stems, water washed and cut into small pieces of 9 ± 3 g of 3–4 cm in diameter and 4 cm in length, homogenized and divided into 27 portions of 180–200 g.

### 2.3. Cooking Treatments

The operative cooking conditions for each thermal treatment (boiling, steam-oven and *sous-vide*) applied to cook the different cauliflower varieties are summarized in Table 1. For boiling (B), one portion of cauliflowers (180–200 g) was dipped in unsalted water (1.5 L) at boiling point in a pot of 18 cm diameter and cooked for 10 and 25 min. For the steam oven (SO) treatment, we used an oven (Bosch, HSG636ES1) purchased in a local distributor (MediaWorld Italy) with steam injection in the chamber (RH% = 100), and the vegetable were cooked at 95 °C at different times (10, 25, 40 min). For *sous-vide* (SV) cooking, cauliflowers were vacuum-packed in a polypropylene heat-resistant (up to 120 °C) bag and submitted to steam oven cooking as reported above. In SO and SV, the oven was not preheated to respect the oven producer program. Each cooking trial was conducted in triplicate. Once cooked, cauliflowers were cooled down to room temperature, knife-cut in small pieces, freeze-dried (Virtis Wizard 2.0 instrument, SP Industries, New York, NY, USA), milled and stored in vacuum pouches at −18 °C. Time and temperature conditions of the three cooking methods were chosen following real household conditions when adopting traditional water boiling or modern commercial ovens, programmed for mild treatments (steam oven and *sous-vide*).

### 2.4. Carotenoids Determination

Carotenoids were extracted in accordance with Biswas et al. [14]. Briefly, freeze dried vegetable (100 mg) was added to acetone (5 mL, 4 °C), kept at 4 ± 1 °C (15 min), vortexed (5 min) and centrifuged (1370 rpm, 10 min, 4 °C), repeating the acetone extraction a second time. The supernatant was filtered (0.45 μm, Sartorius Regenerated Cellulose Membrane), dried, resuspended in 0.5 mL acetone for *Cheddar* samples and 0.25 mL for *Depurple* ones and injected in an Acquity Ultra Pressure Liquid Chromatographic H-class system (Waters Corporation, Milford, CT, USA), equipped with Photodiode Array Detector (PDA). A faster version (20 min rather than 46 min) of the method developed by Chauveau-Duriot et al. [15] was applied using an Acquity column UPLC BEH C18 (2.1 mm × 100 mm, 1.7 μm). The mobile phase was composed by phase A consisting of acetonitrile (75%), dichloromethane (10%) and methanol (15%), and phase B consisting of acetate ammonium in water (0.05 M). Gradient started at 75:25 (A:B) for 10 min, 98:2 (A:B) from 10 to 11 min, 98:2 (A:B) for 20 min. Flow rate was 0.4 mL/min, column oven was set at 35 °C and the sample loading was carried out at 20 °C. PDA analysis was performed at a wavelength of 450 nm using spectrum scanning in the 210–500 nm range. Carotenoids were identified by comparison of retention time and absorbance spectrum with pure standards. Their quantification was performed by external calibration. Good correlation coefficients (R^2^) of 0.999 were obtained in all cases. The instrumental limit of detection (LOD) and quantification (LOQ), calculated with a signal-to-noise ratio of 3:1 and 10:1, respectively, were as follows: neoxanthin, 6 and 21; vioxanthin, 9 and 31, lutein 5 and 16, α-carotene, 5 and 18, and β-carotene, 5 and 18 ng/mL. 

Retinol activity equivalent (RAE) was calculated considering 1 μg retinol for each 6 μg of β-carotene or 12 μg of other provitamin A carotenoids [16].

### 2.5. Tocopherols Determination

Preliminary tests were conducted to optimize tocopherol determination, by direct acetone extraction and saponification as reported by Knecht et al. [17]. A modified saponification procedure was optimized for cauliflower: 0.4 g of powder freeze dried vegetable was added of ascorbic acid (1 g), sodium sulphate (0.1 g), ethanol (20 mL), potassium hydroxide solution 80% (4 mL) and saponified in a water bath (85 °C, 30 min), shaken from time to time. The sample was cooled to room temperature, water was added (12 mL), extracted three times with *n*-hexane (20, 10 and 20 mL) and centrifuged for better separation (2 min, 3600 rpm). The organic phases were pooled, washed four times with water (10 mL), dried with rotavapor at 35 °C and dissolved in *n*-hexane (1 mL) and injected in the UPLC system (UPLC Acquity H-Class, Waters Corporation, Milford, CT, USA) equipped with a Fluorimetric Detector (FLD) and an Ascentis Express HILIC column (15 cm × 2.1 mm, 2.7 μm). An isocratic elution (8 min) of *n*-hexane (95.5%), isopropanol (0.4%) and acetic acid (0.1%) at 0.3 mL/min was performed at 30 °C. FLD was set with an excitation and emission wavelength of 290 and 330 nm, respectively. Tocopherols were identified by comparison of the retention time with pure standards and quantified with external calibration. Standard stock solutions of each tocopherol (α-T, γ-T, δ-T) in isopropanol were prepared in the range of 3.5–100 μg/mL, and good correlation coefficients were obtained for the calibration curves (R^2^ = 0.9836–0.9965) [6]. Limit of detection (LOD) and limit of quantification (LOQ) were as follows: α-tocopherol, 4 and 14, γ-tocopherol, 3 and 11 and δ-tocopherol, 2 and 7 ng/mL. 

Vitamin E was calculated considering only α-tocopherol as proposed by the EFSA NDA Panel [18].

### 2.6. Statistical Analysis

Data are reported as mean values ± standard deviation (SD) of three replicates. Data were analyzed by a one-way ANOVA and Tukey’s mean comparison test at a significance level of *p* < 0.05, as well as principal component analysis (PCA) using R software version 3.5.0.

## 3. Results and Discussion

### 3.1. Impact of Thermal Processing on Carotenoid and Tocopherol Levels

Colored cauliflowers (*Brassica oleracea* L. var. botrytis), such as *Cheddar* (orange) and *Depurple* (purple), are born out of a spontaneous mutation. The presence of the *Or* gene perturbs the normal carotenogenesis and the main carotenoid accumulated is β-carotene, which can reach great levels, namely, 25-fold higher β-carotene levels as compared to white cauliflower, causing it to turn orange [19]. The Purple (*Pr*) gene in cauliflower mutates the pattern of anthocyanin accumulation, coloring the curds and a few other tissues [20]. These colored cauliflowers have recently attracted great attention by consumers due to their high biodiversity, visible not only because of their outer appearance but also their phytochemical profiles [21,22]. Our outcomes highlighted such peculiarities. In fact, considering the raw samples, *Cheddar* and *Depurple* have showed marked differences in terms of fat-soluble antioxidants (Table 2). The total carotenoid content was 10-fold higher in *Cheddar* than in *Depurple*. The most abundant carotenoid in *Cheddar* was β-carotene, followed by lutein, whereas in *Depurple*, lutein was the most plentiful followed by β-carotene. Violaxanthin, neoxanthin and α-carotene were solely detected in *Cheddar*. Our findings agree with those provided by previous research focusing on *Cheddar* and a different purple cauliflower (*Graffiti* cv) [12,19]. However, our results differed in part from those of [11], as lutein and α-carotene in raw *Cheddar* samples were not quantified, and in purple (*Graffiti* variety) only neoxanthin (36.5 mg/kg dried) was found. Additionally, zeaxanthin and cryptoxanthin have been recently found at low levels in raw *Cheddar* [13]. However, such differences could be attributed to the different analytical methodology used for carotenoid extraction and detection. As regards the tocopherol matter, raw *Cheddar* and *Depurple* cauliflowers mainly contained γ-tocopherol (γ-T), followed by α-tocopherol (α-T). δ-tocopherol (δ-T) was solely revealed in *Cheddar* variety, for the first time in such a matrix, to the best of our knowledge. A predominance of γ-T upon α-T was also found in *Cheddar* and *Graffiti* cauliflowers [13].

Aiming to give an overview of the impact of traditional and innovative oven cooking procedures on the carotenoids and tocopherols levels, three types of cooking treatments (boiling, steam oven and *sous-vide*) at different cooking times were applied using two cauliflower varieties. As a result, steam oven (SO) and *sous-vide* (SV) were performed at 95 °C for 10, 25 and 40 min. Boiling was performed at 100 °C for 10 and 25 min. The carotenoids and tocopherols contents (mg/kg dry weight) recorded in raw and processed cauliflowers are reported in Table 2.

**Figure 1:**
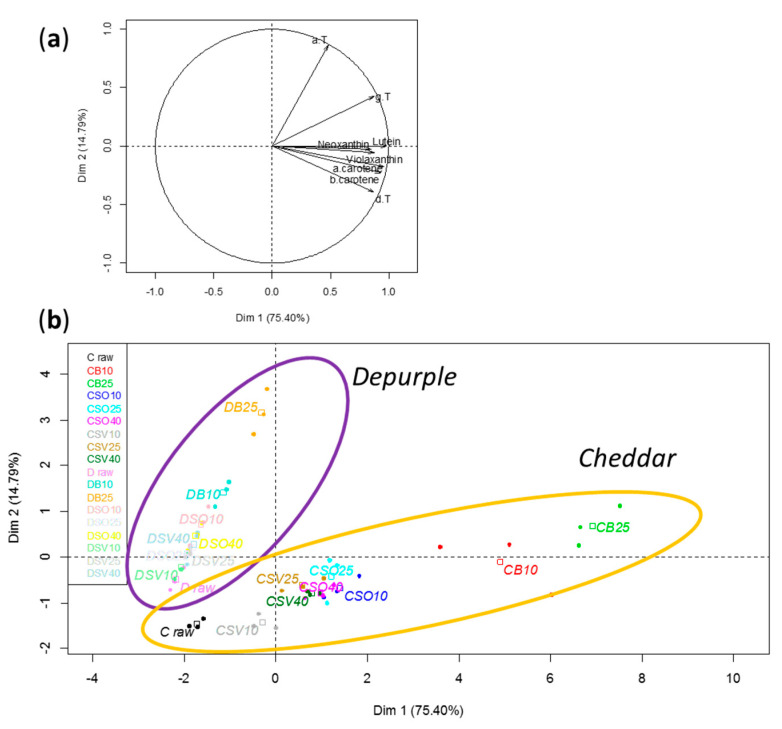
Loading plot of the principal component analysis performed in raw and cooked *Cheddar* and *Depurple* cauliflower by applying eight variables (**a**). Score plot principal component analysis performed in raw and cooked *Cheddar* (C) and *Depurple* (D) cauliflower (**b**).

Following the principal component analysis (PCA) performed on a dataset obtained applying eight variables (neoxanthin, violaxanthin, lutein, α- and β-carotene and α-, γ- and δ-tocopherol), the loading plot (Figure 1a), along the two first principal components (PC1 and PC2), explained 75.44% and 14.77% of the total variance of the cauliflower model, respectively. The score plot (Figure 1b) clearly highlighted that the impact of the tested cooking procedures on liposoluble antioxidants contents was linked to different factors, such as cauliflower genotype, type and time of cooking process. In fact, *Cheddar* and *Depurple* cauliflower samples were split into two distinct clusters. Within each cluster, the samples were grouped according to the cooking test (B, SO, SV) and the time of process (10, 25, 40 min). In both cases, raw, steam oven (SO) and *sous-vide* (SV) samples were grouped together, while boiled samples were clearly distanced. Furthermore, the sample boiled for 25 min can be distinguished from the sample boiled for 10 min.

On these bases, it can be supposed that steam oven and *sous-vide* treatments had a similar impact on the liposoluble antioxidant profile of cauliflowers. Such an impact can be differentiated from that of the boiling process, which also depended on the cooking time.

To corroborate this hypothesis, an ANOVA analysis was also performed. For each compound, within the same cauliflower genotype, significant differences (*p* < 0.05) were highlighted according to the different types of cooking and times. As a consequence, the impact of each investigated cooking practice on each compound for both cauliflower genotypes was investigated and separately discussed in the following subsubsection.

#### 3.1.1. Boiling

Cooking by boiling significantly increases the extractability of all carotenoids and tocopherols, in both cauliflowers. However, the boiling impact on the compound level enhancement depends on the cauliflower variety (matrix effect), kind of compound and time of cooking. The impact was stronger in *Cheddar* rather than in *Depurple*. For example, in *Cheddar* boiled for 25 min (CB25), the total carotenoids and tocopherols contents increased 15-fold and 6-fold, respectively, if compared with the raw sample. Conversely, in *Depurple*, 25 min of boiling (DB25) enhanced the total carotenoids six-fold and the total tocopherol amounts four-fold.

Considering the behavior of each carotenoid compound, the neoxanthin and violaxanthin, which were revealed solely in *Cheddar*, reached significantly highest values for boiling with respect to steam oven and *sous-vide* treatments. Their amounts in CB25 were not statistically different from those detected in CB10. A different performance was seen for α-carotene, the other carotenoid exclusively found in Cheddar. Its highest level was achieved after 25 min of boiling. Diamante et al. [13] also found a higher α-carotene level in *Cheddar* and *Graffiti* boiled for 20 min rather than in that boiled for 10 min.

Lutein displayed a significant increase between 10 and 25 min of boiling, both for *Cheddar* and *Depurple*, whereas 25 min of boiling significantly enhanced the β-carotene amount after only 10 min in *Cheddar*. These findings were partially in contrast with those reported by Diamante et al. [13], showing that a boiling time of up 10 min provoked a decrease of lutein and a retention of β-carotene level in *Cheddar* cauliflower, whereas it led to an increase of both compounds in the *Graffiti* cauliflower.

As regards tocopherol matter, 10 and 25 min of boiling significantly increased the release of all tocopherol compounds (α-, γ- and δ-) in both cauliflowers. It is interesting to notice that the ratio of γ-T/α-T increased from 2 (raw) to around 5 (boiled, 10 and 25 min) in both varieties, suggesting γ-tocopherol is more retained after cooking, as reported by Lee et al. [23] in 5-min boiled broccoli, probably because heat softens plant tissue by cell disruption and may inactivate tocopherol oxidase enzyme.

#### 3.1.2. Steam Oven

Heat transferred to cauliflower by steam (steam oven, 95 °C) had a positive influence on the extractability of both carotenoids and tocopherols. After 25 min of treatment, SO led to a 7.3-fold higher total content of carotenoids compared to raw *Cheddar* cauliflower and to a 2.8-fold increase in *Depurple*. Simultaneously, the total tocopherol content went up by 3.6-fold and 1.5-fold in *Cheddar* and *Depurple*, respectively.

However, the steam oven extractability of carotenoids and tocopherols decreased by two-fold if compared with that of the boiling treatment performed at the same cooking time (i.e., 10 or 25 min). This difference may be the result of a stronger impact of hot water than steam on the microstructure of vegetable tissue. It is widely accepted that the heat may activate the chemical depolymerization reactions of the structural pectin. Such depolymerization reactions cause the disruption of the middle lamella, thus promoting the cell-to-cell separation and the early softening of the vegetable texture. These reactions can progress to such an extent to cause the rupture of the cell wall, promoting the increase of the extractability of the cell contents. The level of the extractability of the compounds, which are compartmentalized at different hierarchical levels in the vegetable tissues, mainly depends on the degree of the extent of pectin degradation reactions, which in turn depends on the specific cooking conditions which include temperature, time of cooking and water availability.

Considering the same cooking time (10 or 25 min), we found that the samples obtained after boiling (CB10, CB25, DB10, DB25) had higher tenderness than the respective steamed samples (CSO10, CSO25, DSO10, DSO25) (data not shown). It is plausible to suppose that steam oven treatment requires more time to reach a similar tenderness to boiled tissues. Accordingly, Borowski et al. [24] studied the effects of steaming and water boiling on firmness, sensory attributes and pectin composition of broccoli. They highlighted that boiling had a more invasive impact on broccoli tissue than steaming, provoking major loss in firmness and the leaching of pectin in boiling water. In fact, boiled broccoli had a lower content of total pectin, protopectin and water-soluble pectin fractions than steamed broccoli.

Furthermore, our results highlighted that the heat transfer efficiency of boiling can be different from that of steaming. In fact, if the boiling impact on the extractability of compounds increased with the cooking time, prolonging the steaming time to over 10 min did not yield additional enhancement of carotenoids and tocopherols levels, except for β-carotene in *Cheddar*, which reached the significantly highest value after 25 min of processing. Peculiar behavior was recognized for violaxanthin. Its amount statistically decreased if the steaming time went up to 10 min. As a result, the violaxanthin content in the *Cheddar* sample that underwent 40 min of steaming (CSO40) was not different from that in the raw sample. It can be supposed the steam oven treatment longer than 10 min provoked the degradation of violaxanthin.

It is plausible to speculate that the texture softening kinetics of different cooking methods involving different heat transfer means (i.e., hot water, saturated steam) are not comparable. Xiao et al. [25] revealed that steaming could cause the formation of a dried layer on the product surface due to the evaporation of water, resulting in a non-uniform heating effect.

Since no other data concerning the effect of steam and *sous-vide* treatments, accomplished in an oven, on carotenoids and tocopherol matters of cauliflowers currently exists in the literature, an extensive comparison of the presented data is difficult.

In agreement with our results, Diamante et al. [13] showed that 10 min of steam cooking performed with a steamer pod was not enough to maximize the extraction of β-carotene in *Cheddar* cauliflowers, whereas it led to a higher release of lutein, α-carotene and all tocopherols than 20 min of steaming. Other authors reported that total carotenoids significantly increased (19%) in oven-steamed broccoli, which is a common technique in Italian cuisine [26], while vessel and oven steam cooking of fresh and frozen broccoli (around 10 min) did not affect the carotenoid content of fresh and frozen counterparts [7]. Steaming (5 min) registered an increment of about seven-fold lutein in uncooked kalian-hybrid-broccoli [27]. Conversely, dos Reis et al. exhibited that 20-min vessel steamed organic cauliflower showed lower values of lutein, β-carotene, total carotenoids and vitamin A than raw cauliflower, and higher values of zeaxanthin, cryptoxanthin and α-carotene [28].

#### 3.1.3. *Sous-Vide*

It is well known that *sous-vide* (or in-pack cooking) is widely applied in restaurants and in catering, because it improves the texture, color and flavor of meat and vegetable foodstuffs due to two factors: the absence of oxygen inside the pack and the mild heat treatment applied [29,30,31,32]. 

Our results show that the ability of the *sous-vide* treatment to release the liposoluble antioxidants from the vegetable tissue was markedly lower than the steam oven treatment, and as a consequence, lower than boiling. In detail, the *sous-vide* oven process enhanced the extraction of the overall carotenoid and tocopherol content mostly in *Cheddar* cauliflower, namely, six-fold and three-fold more than raw cauliflower, respectively (Table 2). A lower extractability was recorded in *Depurple* cauliflower as the total carotenoid content increased by up to 3-fold and 1.5-fold more, respectively.

In order to maximize the β-carotene and lutein extraction, 40 min of *sous-vide* treatment was necessary for both cauliflowers. In *Cheddar* samples, the highest amount of α-carotene was extracted after 40 min of *sous-vide* treatment, while in *Depurple,* α-carotene was below the limit of detection. Differently to steam oven, 10 min of *sous-vide* treatment was not sufficient to maximize the extraction of lutein, in both cauliflowers. Furthermore, no significant increasing trend was observed for α-tocopherol as a consequence of the *sous-vide* treatment of *Depurple* cauliflower. Finally, it was sufficient 10 min of steam oven and *sous-vide* treatments to maximize the extraction of γ-and δ-tocopherol in *Cheddar* whereas the highest amount of γ-tocopherol in *Depurple* was achieved applying sous-vide procedure for a longer time then steam oven.

Although the steam oven and *sous-vide* treatments were performed at the same operative conditions, it can be supposed that there was a different heat-transfer efficiency, resulting in different time–temperature exposure of the processed samples at a given time of cooking, as well as having a different impact on tissue microstructure. It is noticeable that cauliflower tissue is rich in proteins associated with a bilayer of lipids [33]. As a result, *sous-vide* cooking did not reach the same cell disruption enhancement of carotenoids and tocopherols extraction as steaming did. To corroborate this hypothesis, we have found that *sous-vide* samples show the highest stiffness among all the cooked samples (data not shown).

### 3.2. Evaluating the Impact of Thermal Processing on Nutritional Traits of Cauliflowers: Vitamin A and E Contents

**Figure 2:**
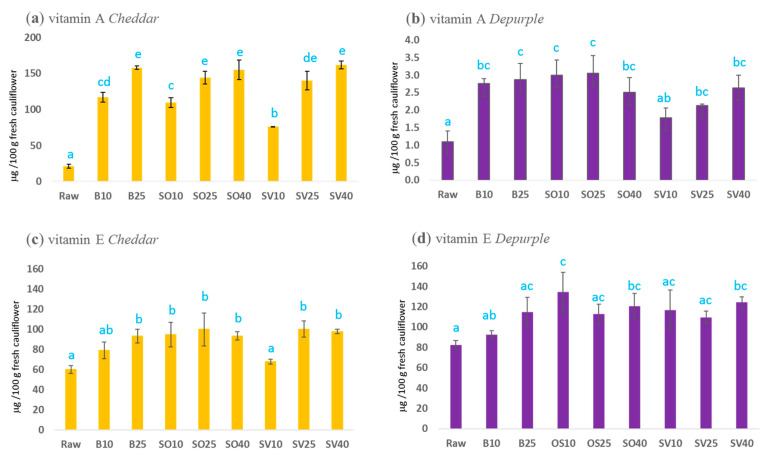
Vitamin A and E contents (μg/100 g fresh cauliflower) in raw and cooked samples. (**a**) Vitamin A quantified in *Cheddar* cauliflower. (**b**) Vitamin A in *Depurple* cauliflower. (**c**) Vitamin E in *Cheddar* variety. (**d**) Vitamin E in *Depurple* cauliflower. Data are expressed as mean of three replicates ± standard deviation (SD). Different superscript letters (a–e) mean significant statistical difference (*p* < 0.05, one-way ANOVA).

Aiming to assess the impact of the tested cooking procedures on nutritional traits of the cauliflowers, vitamin A and E were calculated along EFSA NDA Panel [16,18] for orange and purple cauliflowers submitted to different cooking techniques (Figure 2). As a general rule, all the tested thermal treatments enhanced the extractability of provitamin A and E in *Cheddar* and *Depurple* cauliflower, with some exception in terms of value significance. Differences among traditional (boiling) and mild oven thermal technology (steam oven and *sous-vide*) when expressing the results of vitamin A and E as μg/100 g of fresh vegetable were not observed. This is a consequence of different water uptakes, which lead to apparent changes in α-carotene, β-carotene (retinol equivalents) and α-tocopherol (vitamin E equivalent) concentrations. With reference to raw samples, boiled samples displayed an increment of around 4% of water, while mild oven samples displayed around 1%.

It is of interest to focus on vitamin A content in *Cheddar* cauliflower, as it was a criterion for its fruitful introduction to the market. Figure 2a shows a prolonged time of boiling (25 min), steam oven and *sous-vide* cooking (25 and 40 min) provided the highest contents of vitamin A, which accounted for about 160 μg/100 g fresh cauliflower. Independently of technology, cooking enhanced the availability of vitamin A in any case, and a portion of 200 g of cooked cauliflower provides from 20 to 50% of the reference intakes, set at 750 μg retinol equivalent (RE)/day for men and 650 μg RE/day for women [16]. The impact of thermal treatment in *Cheddar* considering vitamin A is positive. In the same way, vitamin A (Figure 2b) for the *Depurple* variety is enhanced after traditional and mild oven cooking, but the positive trend of time lasting is less evident because of low levels of provitamin A carotenoids, proving in the best case to be 1% (DSO25) of the adequate daily intake of vitamin A.

Considering vitamin E, *Cheddar* (Figure 2c) and *Depurple* (Figure 2d) cauliflowers showed similar levels in all samples (raw and cooked) around a media of 100 μg/100 g wet weight. The lowest values were recorded in raw samples in both varieties; thus, the cooking process did not always enhance the extractability of α-tocopherol in a statically significant way. As in the case of vitamin A, boiling values were flattened to mild oven treatments because of water uptake. In terms of the satisfaction of adequate vitamin E intakes (13 mg/day for men, 11 mg/day for woman) [18], a portion of 200 g colored cauliflower provided, in the best cooking conditions, 1.7% (CSV25) and 2.2% (DOS10).

## 4. Conclusions

It is well known that the heat treatment of vegetable can induce two opposite effects: a reduction of phytochemicals levels as an effect of thermal degradation and/or an increase of extractability of compounds as an effect of matrix softening.

Our results evidenced that all tested procedures led to an increase of carotenoids and tocopherols extractability in cauliflowers. However, the extent of the increase changed according to the cauliflower variety, operative conditions (time and temperature of cooking) and chemical nature of the specific compound. In any case, given the same cooking time and temperature, boiling displayed the highest ability to release all liposoluble antioxidants from the cauliflowers tissue as compared to steam oven and *sous-vide* methods. The latter treatments exhibited similar effects even if the impact of *sous-vide* was more affected by the cauliflower variety than the steam oven. In fact, the *sous-vide* procedure was able to enhance α-tocopherol in *Cheddar* but not in *Depurple* cauliflower.

Although boiling presented the highest ability to release carotenoids and tocopherols, from a nutritional point of view, no differences were revealed among all cooked samples in terms of vitamin A and E contents expressed in μg/100 g fresh vegetable. The higher water content in boiled than in steam and *sous-vide* samples must be considered when the effect of thermal treatment on cauliflower nutritional traits is discussed.

## Figures and Tables

**Table 1:** Cooking treatment and parameters (time, temperature) applied to *Cheddar* (C) and *Depurple* (D) cauliflower varieties.

Cooking Treatment	Equipment	Temperature (°C)	Time (min)	Sample ID ^1^
Boiling (B)	Inox pan	100	10	CB10, DB10
			25	CB25, DB25
Steam oven (SO)	Oven, steam injection (RH% = 100%)	95	10	CSO10, DSO10
			25	CSO25, DSO25
			40	CSO40, DSO40
*Sous-vide* (SV)	Oven, steam injection (RH% = 100%), vacuum sealer machine, food grade plastic poches	95	10	CSV10, DSV10
			25	CSV25, DSV25
			40	CSV40, DSV25

^1^ Sample ID abbreviation: XYn, X = cauliflower variety, Y = cooking treatment, *n* = cooking time.

**Table 2:** Carotenoids and tocopherols content in raw, boiled (B), steam oven (SO) and *sous-vide* (SV) cauliflowers.

	Carotenoids (mg/kg Dry Weight)							Tocopherols (mg/kg Dry Weight)		
	Neoxanthin	Violaxanthin	Lutein	β-Carotene	α-Carotene	∑ Carotenoids	α-T	γ-T	δ-T	∑ Tocopherol
***Cheddar***										
*CRaw*	<LOD	0.2 ± 0.1 ^a^	2.7 ± 0.3 ^a^	17.8 ± 2.4 ^a^	0.2 ± 0.1 ^a^	20.9 ± 2.1 ^a^	8.4 ± 0.6 ^a^	17.9 ± 3.8 ^a^	2.2 ± 0.1 ^a^	28.5 ± 4.4 ^a^
CB10	0.3 ± 0.1	4.6 ± 1.0 ^d^	25.8 ± 3.8 ^d^	180.3 ± 18.5 ^e^	2.5 ± 0.5 ^d^	213.6 ± 21.1 ^e^	20.4 ± 2.3 ^c^	103.3 ± 12.1 ^d^	4.8 ± 0.5 ^c^	128.5 ± 14.3 ^c^
CB25	0.3 ± 0.1	4.5 ± 0.6 ^d^	36.7 ± 0.2 ^e^	268.5 ± 13.4 ^f^	4.0 ± 0.3 ^e^	314.6 ± 13.9 ^f^	26.6 ± 2.8 ^d^	144.1 ± 9.4 ^e^	6.1 ± 0.9 ^d^	176.7 ± 13.1 ^d^
CSO10	0.1 ± 0.0	2.5 ± 0.3 ^c^	12.4 ± 1.1 ^b,c^	99.7 ± 4.8 ^c^	1.3 ± 0.3 ^b,c^	116.1 ± 5.5 ^b,c^	14.5 ± 1.6 ^b^	70.3 ± 7.4 ^b,c^	3.4 ± 0.5 ^b^	88.1 ± 9.4 ^b^
CSO25	<LOQ	1.0 ± 0.1 ^b^	15.5 ± 0.4 ^c^	135.6 ± 7.2 ^d^	1.4 ± 0.1 ^b,c^	153.4 ± 7.1 ^d^	15.7 ± 2.2 ^b,c^	83.7 ± 8.9 ^c^	3.7 ± 0.4 ^b^	103.0 ± 10.9 ^b,c^
CSO40	<LOQ	0.2 ± 0.1 ^a^	14.5 ± 1.9 ^c^	142.7 ± 12.0 ^d^	1.9 ± 0.4 ^c,d^	159.3 ± 12.0 ^d^	14.4 ± 0.7 ^b^	68.9 ± 8.6 ^b,c^	3.5 ± 0.3 ^b^	86.8 ± 9.4 ^b^
CSV10	<LOD	0.3 ± 0.0 ^a^	9.9 ± 1.3 ^b^	67.6 ± 1.2 ^b^	0.9 ± 0.0 ^b^	78.6 ± 0.2 ^b^	9.4 ± 1.2 ^a^	49.7 ± 7.2 ^b^	3.4 ± 0.4 ^b^	62.5 ± 6.3 ^b^
CSV25	<LOD	<LOD	14.9 ± 2.2 ^c^	123.1 ± 12.9 ^cd^	1.3 ± 0.3 ^b,c^	139.3 ± 15.4 ^c,d^	14.7 ± 1.4 ^b^	65.2 ± 8.4 ^b^	3.3 ± 0.5 ^b^	83.3 ± 10.2 ^b^
CSV40	<LOD	0.1 ± 0.0 ^a^	15.9 ± 0.5 ^c^	142.2 ± 2.7 ^d^	1.5 ± 0.2 ^b,c^	159.7 ± 3.1 ^d^	14.4 ± 0.3 ^b^	63.1 ± 4.4 ^b^	3.5 ± 0.6 ^b,c^	81.0 ± 4.7 ^b^
***Depurple***										
*DRaw*	<LOD	<LOD	1.5 ± 0.3 ^a^	0.8 ± 0.2 ^a^	<LOD	2.3 ± 0.5 ^a^	11.1 ± 0.9 ^a^	22.7 ± 4.3 ^a^	<LOD	33.8 ± 5.2 ^a^
DB10	<LOD	<LOD	7.2 ± 0.6 ^c^	3.7 ± 0.2 ^d^	<LOD	10.9 ± 0.7 ^d^	20.8 ± 1.3 ^d^	69.6 ± 2.5 ^d^	<LOQ	90.4 ± 3.8 ^c^
DB25	<LOD	<LOD	9.5 ± 1.4 ^d^	4.4 ± 0.2 ^d^	<LOD	13.9 ± 1.6 ^e^	29.7 ± 2.8 ^e^	104.5 ± 7.5 ^e^	<LOQ	134.1 ± 10.2 ^d^
DSO10	<LOD	<LOD	3.2 ± 0.7 ^b^	2.2 ± 0.2 ^c^	<LOD	5.4 ± 0.8 ^b,c^	17.6 ± 2.1 ^c,d^	50.2 ± 4.9 ^c^	<LOQ	67.8 ± 6.9 ^b^
DSO25	<LOD	<LOD	4.1 ± 0.1 ^b^	2.4 ± 0.3 ^c^	<LOD	6.5 ± 0.3 ^c^	15.0 ± 1.0 ^b,c^	33.3 ± 6.4 ^a,b^	<LOQ	51.4 ± 7.3 ^b^
DSO40	<LOD	<LOD	3.2 ± 0.5 ^b^	2.0 ± 0.3 ^c^	<LOD	5.3 ± 0.8 ^b,c^	16.4 ± 1.5 ^b,d^	43.9 ± 2.1 ^b,c^	<LOQ	60.3 ± 3.6 ^b^
DSV10	<LOD	<LOD	2.1 ± 0.4 ^a^	1.2 ± 0.3 ^a,b^	<LOD	3.3 ± 0.7 ^a,b^	12.8 ± 1.4 ^a,b^	27.8 ± 7.7 ^a^	<LOQ	40.6 ± 8.9 ^a^
DSV25	<LOD	<LOD	3.0 ± 0.4 ^b^	1.7 ± 0.1 ^c^	<LOD	4.7 ± 0.5 ^b,c^	14.5 ± 0.6 ^a,b,c^	34.1 ± 3.6 ^a,b^	<LOQ	48.5 ± 3.9 ^a,b^
DSV40	<LOD	<LOD	4.1 ± 0.3 ^b^	2.2 ± 0.2 ^c^	<LOD	6.3 ± 0.5 ^c^	15.6 ± 2.1 ^a,b,c^	36.2 ± 5.7 ^b^	<LOQ	51.8 ± 7.8 ^b^

Data are expressed as mean of three replicates ± standard deviation (SD). LOD, limit of detection; LOQ, limit of quantification. Different superscript letters (a, b, c) in the same column and cauliflower genotype mean a significant statistical difference analyzed by a one-way ANOVA (*p* < 0.05).

## Data Availability

Not applicable.
